# Macrophage-Derived Immunoglobulin M Inhibits Inflammatory Responses via Modulating Endoplasmic Reticulum Stress

**DOI:** 10.3390/cells10112812

**Published:** 2021-10-20

**Authors:** Xiaoting Gong, Huige Yan, Junfan Ma, Zhu Zhu, Shenghua Zhang, Weiyan Xu, Jing Huang, Xiaoyan Qiu

**Affiliations:** 1 Department of Immunology, School of Basic Medical Sciences, Peking University, Beijing 100191, China; gongxt2014@126.com (X.G.); yanhg@srrsh.com (H.Y.); majunfan2@sina.com (J.M.); jujuonfire@yeah.net (Z.Z.); shenghua1997@bjmu.edu.cn (S.Z.); panziye1206@sina.com (W.X.); 2 NHC Key Laboratory of Medical Immunology, Peking University, Beijing 100191, China; 3 Key Laboratory of Molecular Immunology, Chinese Academy of Medical Sciences, Beijing 100191, China

**Keywords:** macrophage, IgM, Bip, inflammatory response, ER stress

## Abstract

Immunoglobulin (Ig), a characteristic marker of B cells, is a multifunctional evolutionary conserved antibody critical for maintaining tissue homeostasis and developing fully protective humoral responses to pathogens. Increasing evidence revealed that Ig is widely expressed in non-immune cells; moreover, Ig produced by different lineages cells plays different biological roles. Recently, it has been reported that monocytes or macrophages also express Ig. However, its function remains unclear. In this study, we further identified that Ig, especially Ig mu heavy chain (IgM), was mainly expressed in mice macrophages. We also analyzed the IgM repertoire characteristic in macrophages and found that the V_H_DJ_H_ rearrangements of macrophage-derived IgM showed a restricted and conservative V_H_DJ_H_ pattern, which differed from the diverse V_H_DJ_H_ rearrangement pattern of the B cell-expressed IgM in an individual. Functional investigation showed that IgM knockdown significantly promoted macrophage migration and FAK/Src-Akt axis activation. Furthermore, some inflammatory cytokines such as MCP1 and IL-6 increased after IgM knockdown under LPS stimulation. A mechanism study revealed that the IgM interacted with binding immunoglobulin protein (Bip) and inhibited inflammatory response and unfolded protein response (UPR) activation in macrophages. Our data elucidate a previously unknown function of IgM in macrophages that explains its ability to act as a novel regulator of Bip to participate in endoplasmic reticulum stress and further regulate the inflammatory response.

## 1. Introduction

It is well known that immunoglobulin M (IgM) is an ancient molecule that is the first antibody isotype to appear as a specific product of the B cell lineage during evolution [1,2]. Monomer IgM comprises four peptide chains, including two identical heavy chains and two identical light chains. IgM is divided into both membrane and secretory IgM. The membrane IgM appears as a monomer, which is only located on the B cell membrane and responsible for recognizing antigens and then specifically activating B cells. The function of secretory IgM has long been determined only to exert antibody activity, which can be divided into natural IgM and immune IgM [3]. Natural IgM, which shows poly-reactivity and low-affinity, can be spontaneously secreted in an antigen-independent manner to recognize multiple epitopes of different microbial common antigens and exert natural antibody activity. Immune IgM can specifically recognize only one epitope under antigen stimulation and exert adaptor humoral immune function [3].

IgM is a glycoprotein synthesized in the endoplasmic reticulum (ER). To control the correct folding of heavy chains and ensure the fidelity of IgM assembly, the IgM will be glycosylated in the ER and Golgi body via binding its partner molecule, binding immunoglobulin protein (Bip), which is also called Grp78 [4,5]. In recent years, evidence revealed that secretory IgM not only exerted antibody activity but also utilized the sialylated N-linked glycans to inhibit T cell-mediated inflammatory response and play an immune homeostasis role [6]. However, whether intracellular IgM, especially in ER, might have critical physiological functions, like anti-inflammatory, has not been reported.

According to the current classical theory of immunology, IgM has been considered a unique B cells product. However, increasing evidence has proved that non-B cells, such as epithelial cells, cardiomyocytes, renal mesangial cells, and vascular endothelial cells, also produce different isotypes of Ig, including the IgM [7,8]. Moreover, it was found that, like B cell-derived IgM, the epithelial cell-derived IgM also serves as natural antibody activity. The expression and secretion of IgM in epithelial cells were significantly upregulated by CpG ODN, a synthetic analog of bacterial DNA [9,10]. However, it is not yet clear if the IgM produced by non-B cells has other biological functions to date.

Macrophages originate from myeloid hematopoietic cells and are ubiquitous and versatile immune cells in all body compartments [11,12,13]. Macrophages play an innate immune function, which involves the recognition and elimination of pathogens or dead cells by their phagocytosis, and producing inflammatory mediators, including cytokines (i.e., IL-1β, IL-6, and TNF-α) and reactive oxygen species (i.e., nitric oxide) [14,15]. In addition, macrophages are characterized by rapid migration, circulating within almost every tissue, patrolling for pathogens, serving as the first line of defense against infection [13]. So far, it has been considered that macrophages do not produce Ig, and there is no direct association between the above functions of macrophages and Ig. However, it was recently found that myeloid blood cells, such as human monocytes, tumor-associated macrophages (TAM), and acute myeloid leukemia (AML) cells, can express IgG or IgM [16,17,18]. Moreover, AML-derived IgG and IgM can promote the proliferation and migration of AML tumor cells [18,19]. However, it remains unclear if normal macrophages generally express Ig and its significance.

To address if typical macrophages generally express Ig under homeostasis conditions and its significance, in this study, we further identified Ig expression, especially the IgM expression in normal peritoneal macrophages, bone marrow-derived macrophages (BMDM), and macrophage cell line (RAW264.7). Moreover, the intracellular IgM but not membrane IgM was detected in macrophages. IgM knockdown significantly promoted macrophage migration and increased LPS-induced inflammatory response to ER stress via interaction with Bip. The findings suggested that the IgM in macrophages played an anti-inflammatory role via interacting with Bip.

## 2. Materials and Methods

### 2.1. Mice

Balb/c mice between 6–8 weeks of age were obtained from Beijing Vital River Laboratory Animal Technology Co., Ltd. (Beijing, China), and μMT mice (Balb/c background) were from Professor Zhihai Qin (Institute of Biophysics, Chinese Academy of Sciences, Beijing, China). All animal experimental procedures were approved by the Institutional Animal Care and Use Committee for Peking University Health Science Center on 24 February 2017 (LA2017026). All efforts were made to minimize suffering and to reduce the number of animals that were used.

### 2.2. Isolation of Primary Macrophages

Mice were inoculated with 3 mL of 3% sodium thioglycolate via intraperitoneal injection. Peritoneal cells were recovered after 72 h after the sacrifice of mice by lavage with 5 mL of ice-cold phosphate-buffered saline (PBS). The cells were centrifuged at 1000 rpm for 5 min and then were resuspended in Dulbecco’s modified Eagle’s medium (DMEM, Thermo Fisher Scientific, Waltham, MA, USA) containing 10% fetal bovine serum (FBS, Thermo Fisher Scientific, Waltham, MA, USA) and incubated at 37 °C, 5% CO_2_ for 2 h. After removing nonadherent cells by washing three times with pre-warmed PBS, the adherent peritoneal macrophages were added with fresh complete RPMI 1640 (Thermo Fisher Scientific, Waltham, MA, USA) with 10% FBS for subsequent experiments.

Bone marrow cells were isolated from the femur and tibia of 8-week-old Balb/c and μMT mice. To obtain macrophage colony-stimulating factor from fibroblast conditioned medium (FCM, Thermo Fisher Scientific, Waltham, MA, USA), L929 fibroblasts were cultured in RPMI 1640 medium with 10% FBS for 5 days, and the supernatant FCM was collected and centrifuged at 12,000 rpm for 10 min. The centrifuged FCM was stored at −80 °C. Bone marrow cells were cultured in DMEM containing 10% FBS and 20% FCM to generate BMDMs. By the 7th day, all adherent cells had become mature macrophages. The population of CD11b^+^F4/80^+^ adherent peritoneal and BMDMs was then validated and purified by flow cytometry assay (>99% purity).

### 2.3. Cell Culture and Transfection

RAW264.7, a mouse macrophage cell line (ATCC, Rockville, MD, USA), was cultured in DMEM containing 10% FBS, 100 U/mL penicillin, and 100 μg/mL streptomycin (Sigma-Aldrich, St. Louis, MO, USA) at 37 °C in a humidified incubator containing 5% CO_2_.

RAW264.7 cells were transfected with specific siRNA against IgM as follows: siRNA-1, 5′-GUGUGGAAGACUGGAAUAATT-3′ and siRNA-2, 5′-UUAUUCCAGUCUUCCACACTT-3′ synthesized by GenePharma (Shanghai, China). The scrambled siRNA as a negative control (siNC), which is confirmed to have no target in mammalian cells, was also purchased from Shanghai GenePharma (A06001). According to the manual, these siRNAs were transfected into RAW264.7 cells with the Lipofectamine 3000 kit (Thermo Fisher Scientific, Waltham, MA, USA) and scramble siRNA as control. Subsequently, cells were stimulated with lipopolysaccharide (LPS, Sigma-Aldrich, St. Louis, MO, USA).

### 2.4. Protein Extraction and Western Blot Analysis

Total cellular protein was extracted from primary macrophages and RAW264.7 cells using RIPA lysis buffer (10 mM Tris-HCL, pH 7.2, 1% Triton X-100, 1% sodium deoxycholate, 0.1% sodium dodecyl sulfate, 0.15 M NaCl, with fresh protease inhibitor cocktail), and incubated on ice for 30 min. Lysates were then centrifuged at 13,000 rpm for 30 min at 4 °C, and the supernatants were collected for Western blot analysis. The protein samples were separated by SDS-PAGE and transferred onto nitrocellulose membranes (GE Healthcare, Chicago, IL, USA). Membranes were blocked in tris-buffered saline containing 0.1% Tween-20 (TBS-T) and 5% non-fat milk or 5% BSA (for detecting phospho-proteins) for 2 h at room temperature. They were incubated overnight at 4 °C with the appropriate primary antibodies: biotin-conjugated goat anti-mouse IgM (μ chain specific) antibody (VECTOR LAB, Burlingame, CA, USA), and anti-β-actin mAb (MBL, Nagoya, Aichi, Japan). Blots were washed three times for 10 min each with TBS-T and incubated for 1 h at room temperature in the dark with the appropriate IRDye 800- or IRDye 700-conjugated secondary antibodies (LI-COR Bioscience Inc., Lincoln, NE, USA). The signal was detected using the Odyssey Imaging System (LI-COR Bioscience Inc., Lincoln, NE, USA).

### 2.5. Immunofluorescence

Cells grown on cover glasses were washed with ice-cold PBS and fixed with acetone for 5 min. After blocking with PBS with 2% FBS for 30 min, the cells were incubated with rabbit anti-GRP78 antibody (ab21685, Abcam, Cambridge, MA, USA) overnight at 4 °C, and then incubated with AF488 labeled goat anti-mouse IgM mAb (ab150121, Abcam, Cambridge, MA, USA) and FITC-conjugated anti-goat secondary antibody for 30 min at 4 °C. Samples were then washed and mounted in antifade reagent containing Hoechst 33,342 for nuclei staining. After washing, images were observed and acquired using TCS-SP8 STED 3X (Leica Microsystems, Wetzlar, Germany).

### 2.6. Flow Cytometry Analysis

For detection of IgM on the cell surface, the cells were harvested, washed twice with PBS, blocked with 2% FBS in PBS at 4 °C for 30 min, and stained with AF488 labeled goat anti-mouse IgM mAb at 4 °C for 40 min. For assessment of intracellular IgM, the harvested cells were fixed in 4% paraformaldehyde at room temperature for 30 min, washed with PBS, and permeabilized twice with permeabilization buffer (eBioscience, Thermo Fisher Scientific, Waltham, MA, USA). The cells were then centrifuged at 1500 rpm at 4 °C for 5 min. The supernatant was discarded, and the cells were stained with the same antibody as described above. After two washes with PBS, the cells were analyzed on a FACSCalibur flow cytometer using CellQuest software (BD Biosciences, San Jose, CA, USA). Background fluorescence was determined using cells incubated with isotype antibodies.

### 2.7. RT-PCR and Repertoire Analysis of IgM

For the analysis of *IgM* gene expression, RNA was isolated according to the TRIzol LS Reagent manual (Thermo Fisher Scientific, Waltham, MA, USA). The cDNA synthesis was carried out with RevertAid First Strand cDNA Synthesis Kit (Fermentas, Glen Burnie, MD, USA) according to the manufacturer’s instructions. To amplify the aimed genes, the primers in Table 1 were used. For every isolated cell fraction, a quality control PCR was conducted using specific primers for β-actin. No contaminating genomic DNA was detected. To generate the IgM sequences derived from different samples, different barcodes were added to the same sense primers and internal antisense primers to amplify the IgM variable region of different macrophages during the second PCR step. PCR products were separated on 1.5% agarose gel by electrophoresis and extracted using a DNA purification kit (Tiangen, Beijing, China) according to the instruction manual. Concentrations were measured by NanoDrop 2000 spectrophotometer (Thermo Fisher Scientific, Waltham, MA, USA).

### 2.8. Next-Generation Sequencing and Bioinformatic Analysis

Extracted DNA was sent to Novogene Institute (Beijing, China). The amplicons were sequenced on the Illumina HiSeq2500 platform with the MiSeq Reagent Kit V3 (Illumina, San Diego, CA, USA). The raw data (raw reads) in FASTQ format were first processed using Python and custom Perl scripts. Clean data (clean reads) were obtained after removing various reads, including poly-N reads; reads without a 3′ adapter or an insert tag, reads with 5′ adapter contamination; reads with poly-A, T, G, or C; and low-quality reads from the raw data. Moreover, the Q20, Q30, and GC contents of the raw data were calculated. All sequences shorter than 200 bps, having homopolymers of 6 bps barcodes, containing primer mismatches, and with a quality score lower than 19, were removed.

Complementary determining regions (CDR3s) were defined as the segment that encompasses all amino acids flanked by the conserved amino acid sequences Y[YFLI]C at the 3′ end of the V gene segment and [FW]GXGT (X stands for 1 of 20 amino acids) within the J segments. IMGT/High V-QUEST (version 1.7.1) was used for sequence annotation to determine the VDJ genes, CDRs, and junctional modification; characterize nucleotide substitution mutation data for CDRs and framework regions (FRs); and then statistically analyze the usage frequency of VDJ and mutations.

### 2.9. Transwell Assay

The 750 μL DMEM medium with 10% FBS was added into the lower transwell chamber. The transfected RAW264.7 cells in serum-free DMEM medium were seeded onto the upper transwell chamber at a density of 5 × 10^5^/well in 24-well plates. Then, the culture plates were incubated at 37 °C and 5% CO_2_ for 15 h. Afterward, the medium in the chamber was discarded, and the chamber was washed with PBS twice to eliminate the remaining medium. The chamber was fixed with methanol for 30 min, and the cells in the upper chamber were wiped off. After the chamber was air-dried, it was stained for 15 min in 0.1% crystal violet solution, and subsequently washed away. Following air-drying, the chamber was observed under a microscope. Five fields were randomly chosen for each chamber, and images were taken at a magnification of 200×. The number of cells that crossed the membrane was counted and used for the evaluation of the migration of cells. Each test was performed in triplicate.

### 2.10. ELISA of Cytokines

The transfected RAW264.7 cells were treated with LPS (0, 1, 10, 100, and 1000 ng/mL) for 12 h. The culture supernatant from each well was collected at the end of scheduled experiments and used to measure IL-6 and MCP-1 concentration by ELISA according to the manufacturer’s instructions (Thermo Fisher Scientific, Waltham, MA, USA). The absorbance was then measured at 520 nm using a microplate reader.

### 2.11. Glutathione S-Transferase (GST) Pull-Down Assay

IgM cDNA fragments encoding the complete variable domain and first exon of the constant region were PCR-amplified, respectively, and then cloned into a pGEX-4T-2 vector (Amersham Pharmacia Biotech, Buckinghamshire, UK) to produce GST fusion protein. The GST and GST fusion proteins bound to Glutathione Sepharose 4B beads (GE Healthcare, Chicago, IL, USA) were incubated with crude protein extracted from RAW264.7 cells overnight at 4 °C. After washing three times, the Pull-Down mixtures were analyzed by mass spectrum and Western blot analysis.

### 2.12. Statistical Analysis

The data were expressed as the mean ± standard deviation (SD). Statistical differences between multiple groups were analyzed by one-way ANOVA with Tukey’s post hoc test, and two groups were analyzed using the two-tailed Student’s t-test. All statistical analyses were performed using the GraphPad Prism 8 (GraphPad Software Inc., San Diego, CA, USA). *p* < 0.05 was considered statistically significant.

## 3. Results

### 3.1. IgM Expression in Primary Macrophages of Adult Mice and Macrophage Cell Line-RAW264.7

**Figure 1:**
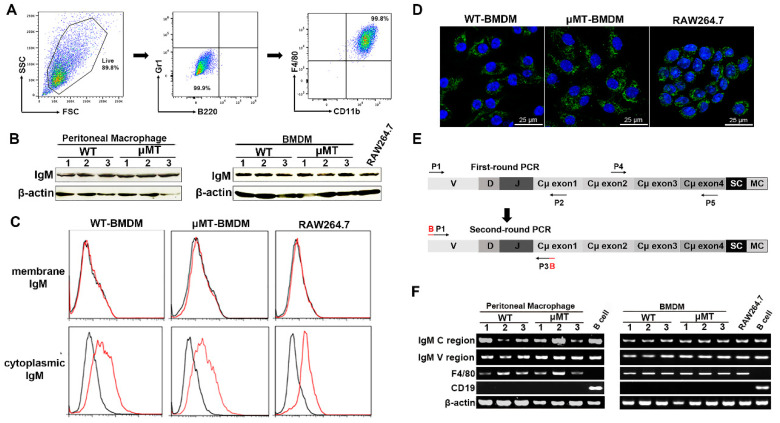
IgM expression in primary macrophages in adult mice and RAW264.7 cell line. (**A**) Purity of the isolated CD11b^+^F4/80^+^ cells validated by flow cytometry. The representative flow cytometry analysis of in vitro differentiated BMDMs from WT and μMT mice demonstrate the purity of 99.9% and absence of B220^+^ B cells/Gr1^+^ granulocytes. (**B**) The mu heavy chain (IgM) was detected in peritoneal macrophages and BMDMs from WT and μMT mice and RAW264.7 cells by Western blot with goat anti-mouse IgM polyclonal antibody. β-actin acted as an internal control. (**C**) Flow cytometry analysis using AF488 labeled goat anti-mouse IgM mAb showed that IgM was localized only in the intracellular space of BMDMs from WT and μMT mice as well as RAW264.7 cells, but not on the plasma membrane of these cells. Black line, isotype control IgG1; red line, anti-mouse IgM. (**D**) Confocal microscopy analysis of BMDMs from WT and μMT mice and RAW264.7 cells using goat anti-mouse IgM polyclonal antibody showed that IgM was present in the intracellular space. DAPI (blue) was used for nuclear staining. Scale bars, 25 μm. (**E**) Diagrams (not to scale) of amplified DNA segments of *IgM* gene variable region and constant region by semi-nested PCR. The arrows indicate the positions of the listed primers in Table 1 used for amplification of the segments. (**F**) *IgM* gene rearrangement and transcriptions in sorted peritoneal macrophages and BMDMs from WT and μMT mice and RAW264.7 cells were analyzed by RT-PCR. The sorted CD19^+^ B cells were used as the positive control. F4/80 as macrophages-specific marker and CD19 as B cells markers were amplified. B, barcodes for primers to differentiate various samples in the next-generation sequencing; MC, membrane component; SC, secreted component; and β-actin as an internal control.

Recent evidence revealed that IgM and IgG were expressed in human CD14^+^ monocytes and macrophages induced by IFN-γ, as well as CD11b^+^ bone marrow macrophages of C57BL/6 mice [16,18]. To further confirm the expression of Ig in macrophages during homeostasis, we first sorted peritoneal CD11b^+^F4/80^+^ macrophages by flow cytometry from both wild-type (WT) Balb/c and μMT mice. The μMT mice are used as a B cell-deficient mouse model, which contains a disruption of the first transmembrane exon of the IgM heavy chain and thus does not express the membrane form of IgM, leading to a lack of mature B cells [20]. The high purify (≥99%) of isolated CD11b^+^F4/80^+^ macrophages and consistent absence of expression of the B cell marker B220 indicated that these cell subpopulations were not attributable to B cell contamination (Figure 1A). Then we further identified if different classes of Ig heavy chain and light chain, such as IgG, IgM, IgA, Igκ, or Igλ can be expressed in these macrophages. As shown in Figure 1B, only the Ig mu heavy chain (IgM) was markedly detected in the primary peritoneal macrophages by Western blot analysis. While other classes of Ig heavy chain and light chain were not detected (data not shown). Next, we induced and obtained differentiated BMDMs from μMT and WT mice and then explored whether the BMDMs also express IgM. We also found IgM expression in the BMDMs (Figure 1B). Of note, the expression level of IgM in BMDMs of μMT mice was consistent with that in WT mice. As expected, we also identified the IgM expression in a mouse macrophage cell line RAW264.7 (Figure 1B). Interestingly, we found that, unlike the B cell-expressed IgM located on the surface of B cells or secreted into extracellular space, the macrophage-expressed IgM exhibited a primarily intracellular staining pattern. In contrast, little or no expression of membranous IgM in BMDMs or RAW264.7 was detected by flow cytometry and confocal microscopy analysis (Figure 1C,D).

Next, we analyzed IgM transcripts by RT-PCR in CD11b^+^F4/80^+^macrophages from both WT and μMT mice. We first used the primers to amplify constant region transcripts of IgM heavy chain. We then found that the constant region transcripts of IgM were markedly demonstrated in these macrophages (Figure 1E,F). The characteristic sequence of functional Ig transcripts is its variable region presenting typical V (D) J rearrangement. As expected, we also determined the IgM transcripts with a typical VDJ rearrangement of the variable region in the primary peritoneal macrophages and BMDMs from either WT or μMT mice, as well as in RAW264.7 cells (Figure 1E,F). The results indicated that macrophages expressed IgM, mainly located in intracellular compartments but not on the cell surface.

### 3.2. The Repertoire of IgM Heavy Chain Variable Region Sequences Displays Restricted Diversity in Macrophages

**Figure 2:**
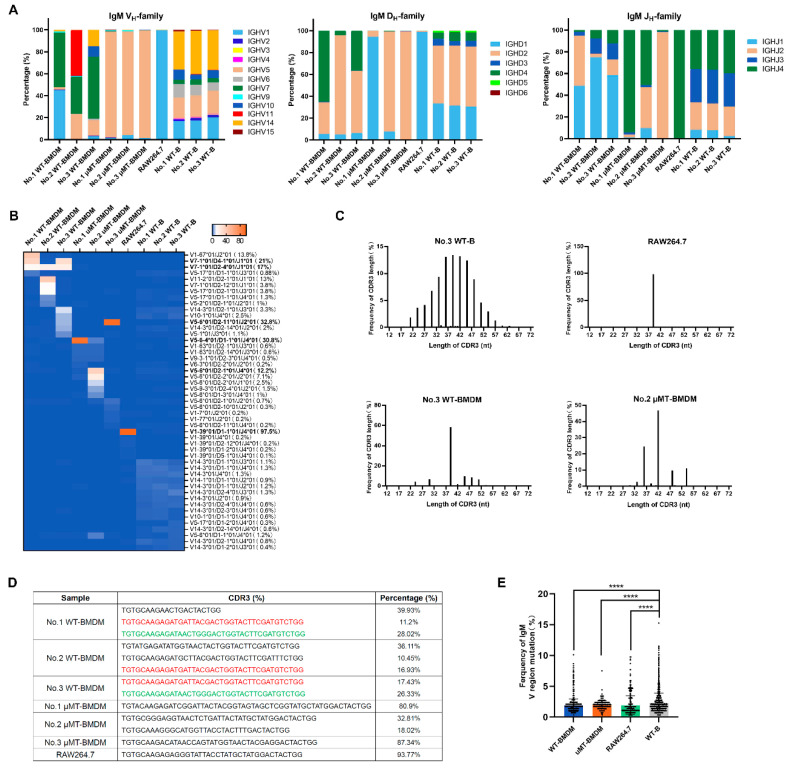
Repertoire features of IgM variable region sequences from murine macrophages. (**A**) IgM V_H_, D_H_, and J_H_ gene family usage profiles of BMDMs from WT and μMT mice and RAW264.7 cells compared with B cells. The frequency (%) of V_H_ family genes (left), D_H_ family genes (middle), and J_H_ family genes (right) expressed by macrophages and B cells, respectively, from three different individuals, demonstrated the IgM derived from macrophages differed significantly from that of the B-derived IgM. (**B**) Distribution of restricted V_H_DJ_H_ rearrangement patterns of BMDMs from WT and μMT mice and RAW264.7 cells compared with B cells. The percentage of different rearrangement patterns is given in parentheses. (**C**) Length variant analysis of CDR3 demonstrates the constitutive expression of individual-specific IgM variable heavy-chain repertoires in B cells. Non-normalized CDR3 length spectra typing for BMDMs from WT and μMT mice and RAW264.7 cells. (**D**) Sequential CDR3 repertoire analyses in BMDMs from WT and μMT mice and RAW264.7 cells. The common sequences were colored in red or green consistently. (**E**) Mutation frequencies of IgM variable region in BMDMs from WT and μMT mice and RAW264.7 cells compared with B cells. Abundance was first normalized by region length and then by total number of cleaned, productive reads in each respective data set, and multiplied by 100 to attain percent abundance. **** *p* < 0.0001.

It has been reported that rearranged IgM variable region was expressed in a small macrophage subfraction, which showed markedly restricted Ig repertoires compared to normal B cells [16]. In this study, we further analyzed the rearranged sequences of IgM V_H_DJ_H_ in BMDMs of three WT and three μMT mice, as well as in the RAW264.7 cells, by next-generation sequencing. The B cell-derived IgM served as a positive control. We obtained 8911 reads of IgM transcripts in BMDMs of WT mice, 3175 reads of IgM transcripts in BMDMs of μMT mice, and 8295 reads of IgM transcripts in RAW264.7 cells. The sequence analysis results demonstrated that all IgM transcripts showed a typical VDJ rearrangement pattern, and above 90% of V_H_DJ_H_ rearrangements from macrophages showed a functional rearrangement (Table 2). Next, we analyzed the usage of the V_H_, D_H_, and J_H_ families in macrophages compared to the corresponding B cells. All V_H_, D_H_, and J_H_ gene families were distributed in B cells, respectively, according to previously published numbers for normal B cells [21]. Among these sequences, we have noted that several V_H_ families, such as V_H_1, V_H_5, and V_H_14, are most highly selected in B cells, which are consistent with another study [22]. At the same time, the macrophage-derived IgM mainly selected V_H_7; D2, D4, and J_H_1 in BMDMs of WT mice, and BMDMs of μMT mice preferentially selected with V_H_5; D1, D2, and J_H_2; and J_H_4. The RAW264.7-derived IgM only used V_H_1, D1, and J_H_4 (Figure 2A). Of note, several identical patterns of V_H_DJ_H_ rearrangement, such as V7-1*01/D4-1*01/J1*01 (21%, 1810/8620 indicated as the number of biased sequence vs. the total number of all productive sequences derived from BMDMs of WT mice) and V7-1*01/D2-4*01/J1*01 (17%, 1465/8620), were observed in BMDMs of WT mice. Still, the V5-6-4*01/D2-11*01/J2*01 (32.8%, 1041/3175), V5-6-4*01/D1-1*01/J4*01 (30.8%, 978/3175), and V5-6-4*01/D2-1*01/J4*01 (12.2%, 387/3175) frequently showed in BMDMs of μMT mice (Figure 2B). Interestingly, a unique oligoclonal rearrangement pattern, with V1-39*01/D1-1*01/J4*01 (97.5%, 7747/7946) was displayed in RAW264.7 cells (Figure 2B). Importantly, some identical V_H_DJ_H_ rearrangements such as V7-1*01/D2-4*01/J1*01 were detected in the BMDMs among different WT individuals, even the V5-6-4*01/D2-11*01/J2*01 was found between WT and μMT mice. All the above results revealed that macrophage-derived IgM repertoires showed restricted diversity and conservativeness.

Subsequently, we compared the complementarity determining region 3 (CDR3) length of BMDM-derived IgM with B cell-derived IgM. As expected, the CDR3 in different B cells from an individual showed different lengths (presented as spectra type plots). However, most CDR3 in different BMDMs from an individual showed the same length because there are many high frequencies of clonotype of shared VDJ rearrangements in an individual, even among different individuals of either WT or μMT mice (Figure 2C). To further assess whether the dominant CDR sequence of IgM VDJ rearrangements also existed in macrophages, we compared CDR3 sequences among all the individuals. The most frequent CDR3 sequences were exhibited in BMDMs and RAW264.7 cells as a combinatorial and individual-specific pattern. Significantly, identical sequences were found in the BMDMs from different WT individuals but not between WT and μMT mice (Figure 2D), suggesting the hierarchy of prevalent clonotypes was broadly conserved in different individuals.

We also analyzed mutation frequencies of the IgM variable region sequences derived from macrophages. Interestingly, the overall number of mutations in the IgM variable region genes from BMDMs of both WT and μMT mice showed a lower mutation frequency than those in B cells (Figure 2E). Thus, the differences in the IgM repertoire diversities between macrophages and B cells were also based on distinct abilities for mutation of IgM variable region.

### 3.3. Knockdown of IgM Enhanced the Migratory Ability of Macrophages through Activating Src/FAK Axis

**Figure 3:**
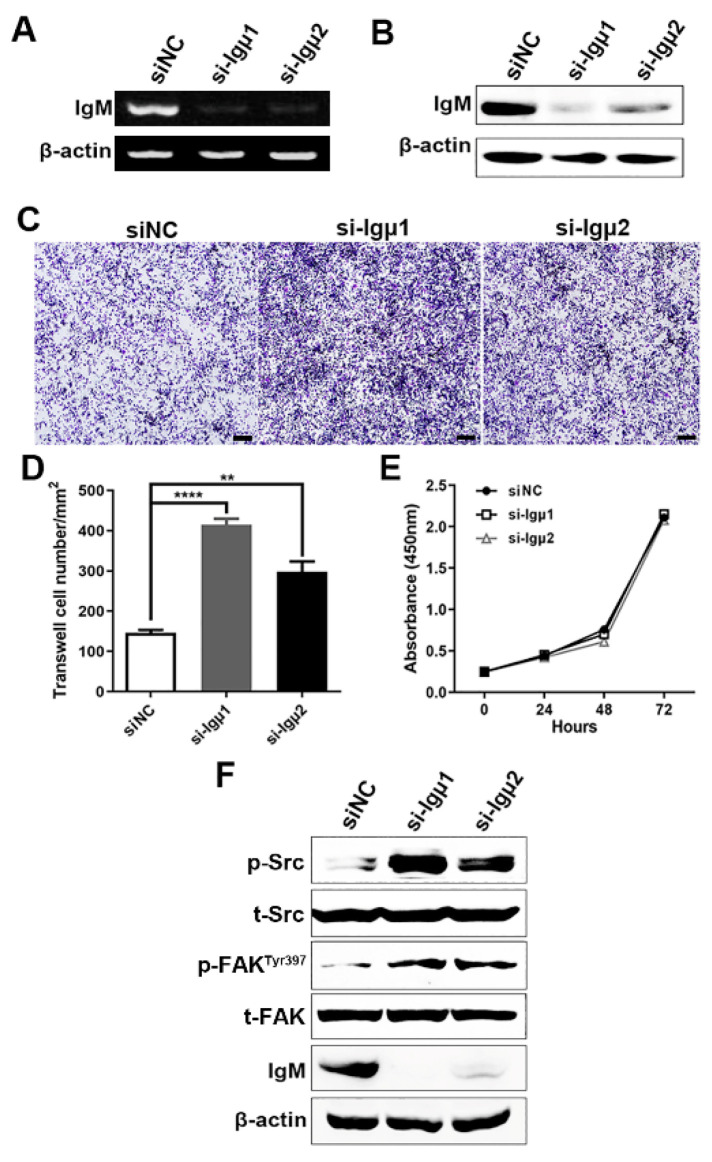
Knockdown of IgM enhanced the migratory ability of murine macrophages through the Src/FAK axis. IgM expression was determined by RT-PCR (**A**) and Western blot (**B**) in the RAW264.7 cells by specific siRNA against IgM (si-Igμ) and control siRNA (siNC). (**C**) The effects of IgM siRNA on cell migration were analyzed by transwell assay, showing that IgM knockdown promoted the migration ability of RAW264.7 cells. Scale bar = 200μm. (**D**) Data are expressed as the mean ± SD of migrated cells number per five fields. (**E**) The effects of IgM siRNA on cell proliferation were analyzed by CCK8 assay. The data are presented as mean ± SD (*n* = 3). (**F**) Western blot analysis of Src/FAK signaling after IgM knockdown for 24 h. β-actin as an internal control. ** *p* < 0.01, **** *p* < 0.0001 vs. control siRNA.

Based on IgM expression and characteristics of IgM transcript in RAW264.7 cells, which was consistent with primary macrophages, it was chosen as a cell model for further analysis. To evaluate the effects of IgM, RAW264.7 cells were transfected with specific small interfering RNA (siRNA) against IgM constant region. After the effective knockdown of IgM in RAW 264.7 cells was confirmed by RT-PCR and Western blotting assays (Figure 3A,B), we found that RAW264.7 cells gradually became spindle-shaped (data not shown). Moreover, knockdown of IgM remarkably promoted RAW264.7 cell migration using transwell assays (Figure 3C,D) but had no significant effects on cell viability and proliferation through CCK8 assay (Figure 3E).

It is well known that Src/FAK axis played a pivotal role in macrophage mobilization [23]. Thus, to ascertain the involvement of the Src/FAK signaling pathway in IgM-mediated macrophage migration, the phosphorylated statuses of Src and FAK in RAW264.7 cells were examined. As expected, significant increases in phosphor-Src, -FAK at Tyr 397 were observed in RAW264.7 cells after the knockdown of IgM. However, total Src and FAK were unaltered (Figure 3F). Thus, the results indicated that macrophage-derived IgM indeed inhibited macrophages’ migration via Src/FAK signaling.

### 3.4. Knockdown of IgM Promoted Pro-Inflammatory Response of Macrophages through Secreting Pro-Inflammatory Mediators

**Figure 4:**
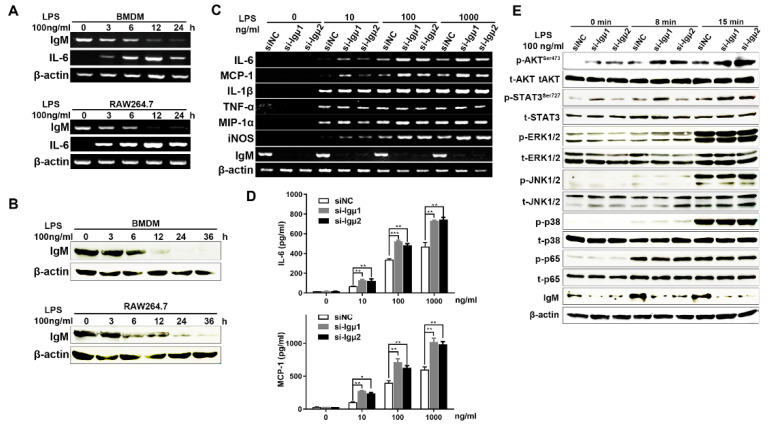
IgM knockdown promoted a pro-inflammatory effect in LPS-induced RAW264.7 macrophages through Akt signaling. The upregulated expression of IgM in BMDMs and RAW264.7 cells stimulated with 100 ng/mL LPS for 0, 3, 6, 12, and 24 h by RT-PCR (**A**) and Western blot (**B**). (**C**) Cells were transfected with IgM siRNA (si-Igμ) and control siRNA (siNC) for 36 h and treated with various concentrations of LPS (0, 1, 10, 100, and 1000 ng/mL) for an additional 3 h. The pro-inflammatory cytokines, including IL-6, MCP-1, IL-1β, TNF-α, MIP-1α, and iNOS, were determined by RT-PCR. IgM acted as positive control and β-actin as an internal control. (**D**) Cells were transfected with IgM siRNA (si-Igμ) and control siRNA (siNC) for 36 h and treated with LPS (0, 1, 10, 100, and 1000 ng/mL) for an additional 12 h. The production of IL-6 and MCP-1 cytokines was measured by ELISA kit using the microplate reader. The data are presented as means ± SD (*n* = 3). (* *p* < 0.05 and ** *p* < 0.01, *** *p* < 0.001 vs. siNC control group). (**E**) Cells were transfected with IgM siRNA (si-Igμ) and control siRNA (siNC) for 36 h before exposure to LPS (100 g/mL) for 8 and 15 min. Enhanced effects of IgM knockdown on phosphorylation of AKT at Ser 473 and STAT3 at Ser 727 in LPS-induced RAW264.7 macrophages were shown by Western blot analysis, but no obvious effect on phosphorylation of ERK1/2, JNK1/2, p38, and p65 was shown. p, phosphorylated protein; t, total protein. IgM acted as positive control and β-actin as internal control.

On account of the macrophages treated with LPS, which had characteristics of activation and are pro-inflammatory, we firstly explored whether IgM is involved in LPS-stimulated macrophage activation in vitro. Both primary BMDMs and RAW264.7 cells were treated with LPS, and then we analyzed the IgM expression level at different time points. The results showed that the IgM expression was significantly decreased in a time-dependent manner in both LPS-stimulated BMDMs and RAW264.7 cells following LPS stimulation for 12 h (Figure 4A,B). The observation indicated that macrophage-derived IgM might play an essential role in the LPS-induced macrophage pro-inflammation process.

Since activated macrophages release pro-inflammatory mediators, such as IL-6, MCP-1, and NO, we further investigated the role of IgM in LPS-induced macrophage inflammation response. As shown in Figure 4C, knockdown of IgM significantly upregulated the levels of IL-6, MCP-1, and iNOS mRNA induced by LPS in a dose-dependent manner. Meanwhile, IL-6 and MCP-1 protein levels increased dramatically after IgM knockdown with LPS stimulation using ELISA assay (Figure 4D). These results suggested that IgM exerted anti-inflammatory activity via the inhibition of NO production and pro-inflammatory cytokines IL-6 and MCP-1 in macrophages.

To determine the downstream signaling pathways involved in the IgM-mediated anti-inflammation effect stimulated with LPS, we first investigated the involvement of TLR4-mediated PI3K-Akt, MAPK, and NF-κB pathways in macrophages. Since TLR4 is a receptor that binds to LPS, activates its downstream signaling pathways, and induces various inflammatory responses. The RAW264.7 cells were treated with LPS for 0, 8, and 15 min and then analyzed by Western blot analysis. As shown in Figure 4E, the elevated level of Akt, ERK1/2, JNK, and p38 phosphorylation were observed after LPS stimulation, while knockdown of IgM only upregulated phosphorylation of Akt at Ser473, playing a critical role in the inflammation pathway, but had no effect on MAPK signaling pathway in LPS-induced macrophage. In addition, IgM knockdown also upregulated the phosphorylated level of STAT3 at Ser727, which is a vital regulator of the inflammatory response, and its activation promotes the expression of inflammatory cytokines, including IL-6 and MCP-1. In addition, we also investigated the LPS-induced NF-κB pathway and found that the phosphorylation of p65 remained unchanged when IgM was knocked down (Figure 4E). Overall, these results indicated that the anti-inflammatory effect of IgM in macrophages might be, in part at least, dependent on Akt and STAT3 phosphorylation.

### 3.5. IgM Located in ER and Interacts with Bip in Macrophage

**Figure 5:**
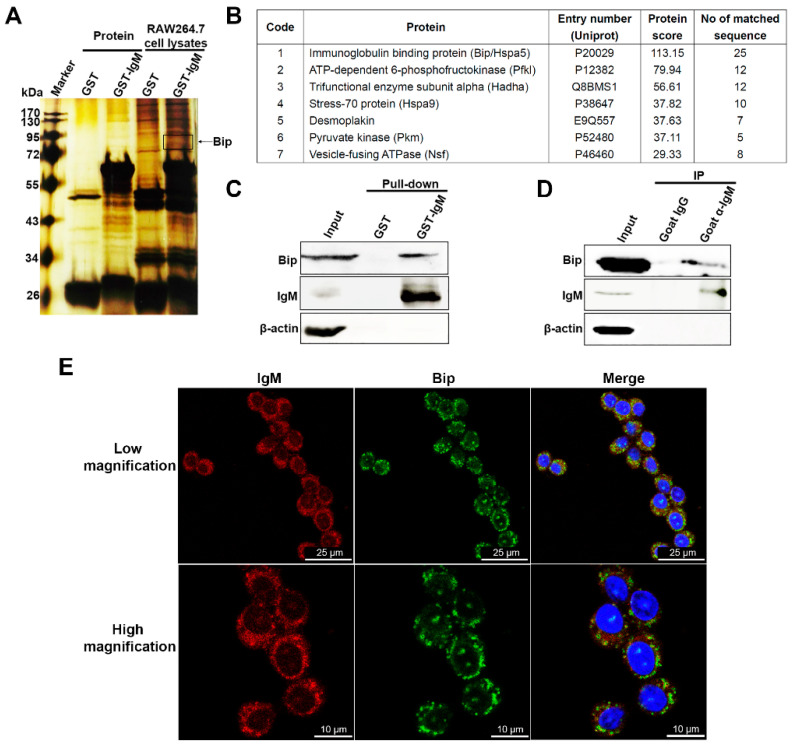
IgM interacted with Bip in RAW264.7 cells. (**A**) Whole cell lysate derived from RAW264.7 cells was used to pull down with the same amount of GST-IgM recombinant protein or GST protein as control, respectively. The complexes were separated on SDS-PAGE gels. After silver staining, the differential bands or dots were analyzed by MS. Asterisks and circles indicated for the differential band of Bip. (**B**) Candidates of IgM-associated proteins were identified by liquid chromatography-mass spectrometry. Only part of the identified proteins was shown after removal of repeated proteins and proteins related to the heavy or light chains of the antibody. (**C**) Western blot analysis to determine the interaction between IgM and Bip in GST-pull down assay. (**D**) The interaction between endogenous IgM and Bip was shown in the coimmunoprecipitation assay. The cell lysate of RAW264.7 cells was immunoprecipitated with goat anti-IgM antibody and goat IgG as control. Input, RAW264.7 cell lysate. (**E**) Confocal microscopy analysis of IgM (red) and Bip (green) in RAW264.7 cells using goat anti-mouse IgM polyclonal antibody and rabbit anti-mouse Bip antibody. DAPI (blue) was used for nuclear staining. Scale bars, 25 μm (Low magnification) and 10 μm (High magnification).

To further explore the anti-inflammatory mechanism of macrophage-derived IgM, we first constructed an expression vector containing GST and IgM heavy chain, which had a unique V1-39/D1-1/J4 rearrangement pattern expressed by RAW264.7, to find the potential interacting protein of IgM. Next, the specific GST-IgM prokaryotic protein and GST protein as control were expressed and purified. They then were used to find and identify the interacting protein of IgM using RAW264.7 cell lysate by pull-down assay and mass spectrometry. Some differential proteins were pulled down in GST-IgM fusion protein but not GST protein alone (Figure 5A). By mass spectrometry and Western blot analysis, the differential proteins were identified to be an immunoglobulin binding protein (Bip; also known as Grp78 and Hspa5), ER-resident chaperone, and heat shock protein (Hsp70; also known as Hspa9) (Figure 5B,C). Moreover, we also confirmed the presence of IgM-Bip complexes in RAW264.7 cells by coimmunoprecipitation assay (Figure 5D). In addition, we also revealed that IgM co-localized with Bip in RAW264.7 (Figure 5E).

### 3.6. IgM Regulating Bip Expression via IRE1α-Mediated XBP1 mRNA Splicing Is Necessary for ER-Stress-Induced Macrophage Activation

**Figure 6:**
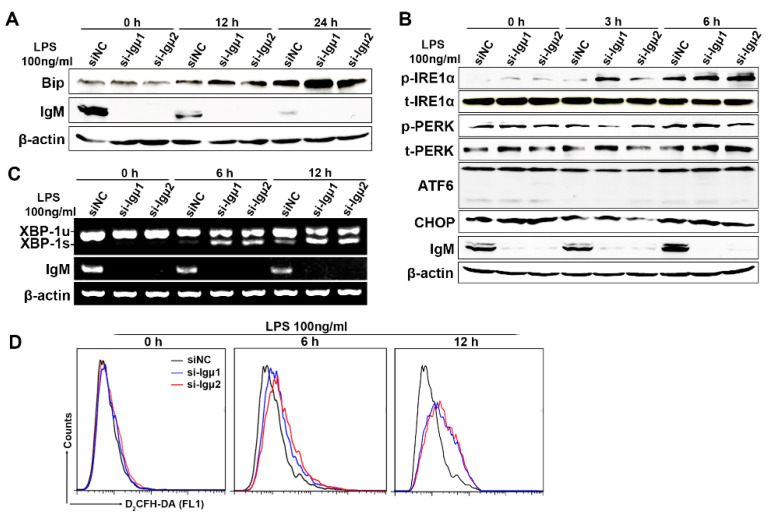
IgM knockdown promoted the IRE1α branch of the ER stress pathway through regulating Bip expression. RAW264.7 cells were transfected with IgM siRNA (si-Igμ) and control siRNA (siNC) for 36 h before exposure to LPS (100 g/mL) for the indicated times. (**A**) Intracellular Bip protein expression was upregulated after IgM knockdown by Western blot analysis. β-actin acted as an internal control. (**B**) Enhanced effects of IgM knockdown on phosphorylation of IRE1α but not PERK, ATF6, and CHOP were shown by Western blot analysis. (**C**) RT-PCR analysis showing the Expression of XBP1u mRNA and XBP1s mRNA. IgM as positive control and β-actin as an internal control. (**D**) Representative flow cytometric histograms showing ROS production measured by the H2DCF-DA probe in RAW264.7 cells. blue line, si-Igμ1; red line, si-Igμ2; black line, siNC as control siRNA.

Bip is a major ER chaperone and is suggested to act as a primary sensor in activating the unfolded protein response (UPR) [24]. During unstressed conditions, Bip binds to and inhibits the three ER-proximal UPR transmembrane proteins: pancreatic ER kinase (PERK), inositol-requiring transmembrane kinase/endonuclease 1 (IRE1), and activating transcription factor 6 (ATF6). ER stress is averted as long as newly synthesized proteins fold correctly and transit into the ER lumen. When unfolded proteins accumulate during stress, Bip is recruited away from ATF6, IRE1, and PERK to activate the different UPR signaling axes [25,26]. So far, it is only known that Bip combined with IgM heavy chain in the ER helps IgM folding and glycosylation as a chaperone molecule [27], but whether IgM heavy chain might affect the activity of Bip as a UPR sensor has not been reported. To address if the IgM might be involved in UPR via interacting with Bip, we examined whether IgM regulated the expression of Bip in an ER stress model induced by LPS. We found that knockdown of IgM significantly enhanced the level of LPS-induced Bip protein in a time-dependent manner (Figure 6A). To further elucidate if IgM was involved in any UPR responses to promote macrophage activation, we examined the effect of IgM on phosphorylation of PERK and IRE1α, as well as cleavage of ATF6, in RAW264.7 cells. It can be seen from Figure 6B that LPS stimulation significantly induced the phosphorylation of IRE1α, and knockdown of IgM further increased the level of phosphorylated IRE1α induced by LPS in a time-dependent manner. However, knockdown of IgM had no apparent effect on the level of phosphorylated PERK and downstream CHOP, as well as ATF6 cleavage. Since IRE1α phosphorylation stimulates endoribonuclease activity, which splices mRNA of XBP1 to form a potent transcriptional activator, we further examined whether IgM inhibited the mRNA level of spliced XBP1 (XBP1s). As shown in Figure 6C, knockdown of IgM indeed enhanced the mRNA level of spliced XBP1 induced by LPS in a time-dependent manner. Since prolonged ER stress promotes ROS release to enhance the killing effect of macrophages, and ROS has also been reported to regulate the activation of XBP1 [28], we thus investigated whether IgM knockdown underwent ER stress due to increased oxidative stress. We found that the knockdown of IgM significantly upregulated ROS (Figure 6D). In summary, IgM knockdown promoted the IRE1α branch of the ER stress pathway through regulating Bip expression, thereby mediating macrophage activation.

## 4. Discussion

In the present study, we extended previous findings and demonstrated IgM expression in macrophages of mice under hemostasis conditions, especially the macrophages of B-cell deficient mice (μMT mice), and further revealed that the repertoire features of IgM variable region sequences exhibited individual-specific effects using next-generation sequencing. More importantly, we identified for the first time that IgM interacted with Bip to inhibit ER stress via suppressing IRE1α-mediated XBP1 mRNA splicing, thereby inhibiting the migratory ability of macrophages and decreasing LPS-induced pro-inflammatory response in macrophages.

The classical immunological theory has long defined that only B cells can produce Ig among the blood cells. In contrast, myeloid blood cells, such as monocytes and macrophages, do not produce Ig. Thus, as long as Ig-expressing cells are found clinically, they are classified as B cells. Increasing shreds of evidence have proved that myeloid blood cells also produce Ig. This fact was first uncovered that AML cells expressed IgG, IgM, and IGK, all of which promoted the occurrence and development of AML. For example, AML-derived IgG and IGK promoted the migration of AML cells, and their expression predicted poor prognosis of AML patients [19,29]. IgM was also involved in the proliferation of AML cells [18]. Recently, some studies have found that Ig was expressed in tumor-infiltrating macrophages and ex vivo differentiated macrophages [16,17]. Consistent with this, in our present study, evidence for the expression of the Ig mu heavy chain (IgM) was further confirmed in macrophages of both B-cell-deficient and WT mice under hemostasis, as well as a macrophage cell line. Interestingly, we demonstrated the presence of the mu heavy chain alone without the light chain, including κ and λ chain, in primary macrophages and RAW264.7 cells, suggesting that IgM derives from mouse macrophages perhaps as a free heavy chain by itself to exert bioactivity in the macrophages.

As is well known, a diverse repertoire of B-cell-derived Ig contributes to immunity against a vast number of potential pathogenic threats. The generation of Ig diversity begins with Ig variable region assembly from V, D, and J gene segments to form the antigen recognition piece of the B cell receptor (BCR), which was initially expressed as IgM on the cell membrane and then secreted into extracellular space [30]. However, it has been revealed that non-B cell-derived Ig, including the IgM, usually displays non-antibody activities involved in cell survival, adhesion, and proliferation [7,9,10,31,32]. Therefore, the repertoire of non-B-cell derived Ig exhibits restricted diversity compared with that of B-cell-derived Ig. The restricted diversity reflected that Ig from the same lineage often displays several dominant VDJ rearrangement patterns and even shares them among different individuals. The characteristics suggest that the function of non-B cell-derived Ig might be different from that of B cell-derived Ig, and these dominant VDJ rearrangement patterns shared by different individuals might act on similar pathways. In this study, we used next-generation sequencing to analyze the repertoire characteristic of macrophage-derived IgM and found that the Ig repertoires of macrophage showed biased V_H_, D_H_, and J_H_ family usage. Moreover, similar to the IgM repertoire in other non-B cells [16,17,18,33], the macrophage-derived IgM in an individual often displays several dominant VDJ rearrangement patterns. However, only a few VDJ rearrangement patterns of macrophage-derived IgM were shared by different individuals, which suggested that the different usage of VDJ rearrangement patterns may be related to the ontogeny of macrophages. Interestingly, a unique oligoclonal rearrangement pattern was displayed in RAW264.7 cells, and it is a real possibility that a genomic VDJ rearrangement occurred once prior to the establishment of the RAW264.7 cell line. The specific significance needs to be further revealed. It has also been reported that myeloid cell-derived IgM shares the restricted or biased usage of specific sequences under certain selection pressure due to neoplastic conditions compared to normal B cells [18]. It is worth noting that the Ig repertoires from tumor-associated macrophages (TAM) and circulating monocytes in glioblastoma patients revealed a negative correlation to the tumor volume, which could not be detected in the Ig repertoires of the patients’ B lymphocytes [17]. Thus, macrophage-derived IgM might be a novel molecule with a unique structure and function, revealing yet unrecognized host defense mechanism in macrophages.

Meanwhile, IgM on the B cell surface responsible for the negative selection of B cells facilitates B cell development, recognizes an antigen, and enhances the humoral immune response to the antigen. The secreted IgM provides the first line of defense against pathogens, thereby maintaining tissue homeostasis [1]. In addition, it has been reported that the secreted IgM exerts an anti-inflammatory response by suppressing the activation of T cells [6]. However, intracellular IgM has never been reported to exert this function to date. In this study, we found that silencing IgM significantly promoted cell migration and activation of the FAK/Src signaling pathway, which involves cell migration. It has been reported that expression or activation of Src, a cellular nonreceptor tyrosine kinase downstream of integrin signaling, could reflect the migratory ability of macrophages [34]. As a substrate of Src, FAK serves as an adaptor that connects integrins with the actin cytoskeleton. Upon integrin activation, FAK is phosphorylated at tyrosine residues Tyr397, activating the binding site Src [35]. Those results hint that IgM might inhibit macrophage migration via the FAK/Src axis.

More importantly, mechanism research reveals that IgM is located in ER and interacts with Bip, the upregulation of which is the most used marker for UPR signaling activation [36]. Bip was initially named for its binding to Ig heavy chains as a vital chaperone molecule involved in the protein folding and assembly in ER [4]. When a newly synthesized Ig heavy chain does not bind with the light chain in ER, the Ig heavy chain will couple with Bip. Next, Ig heavy chain is retained in the cytoplasm but not transported to the extracellular matrix or cell membrane. It has been reported that the variable region and the first constant region of Ig heavy chain are key regions mediating this binding with Bip [37]. Moreover, we identified the interaction of Bip and IgM with a unique V1-39/D1-1/J4 rearrangement pattern derived from RAW264.7 cells. Therefore, we conjecture that limited VDJ rearrangements of IgM might bind with BiP in macrophages to exert bioactivity. However, it remains unclear if IgM regulates Bip-mediated function. This study first found that ER stress induced by LPS stimulation resulted in reduced IgM expression. At the same time, IgM knockdown markedly increased the expression of Bip and inflammatory mediators in macrophages, such as MCP1 and IL-6, as well as iNOS. The findings suggest that IgM might play a negative mediator role for ER stress in macrophages. It is well known that there are three key UPR signal activators—PERK, IRE1α, and ATF6—which give rise to separate branches of the response [38,39]. In the absence of ER stress, the PERK, IRE1α, and ATF6 proteins are sequestered in inactive complexes with Bip and maintain the downstream transduction signals. During ER stress, the accumulating unfolded-protein species preferentially bind Bip to dissociate it from IRE1α, PERK, and ATF6 complexes, resulting in autophosphorylation of IRE1α and PERK, and mobilization of ATF6 to the Golgi for activation [25,38]. The activated IRE1α splices XBP1u mRNA into XBP1s mRNA, from which protein XBP1s is translated and then translocated into the nucleus during the early phase of ER stress [40]. The splicing XBP1s binds to the promoters of IL-6 and TNF-α and promotes their transcription and expression [41]. We further address if IgM is involved in any of the UPR responses to activate macrophage activation. The results revealed that knockdown of IgM only promoted the phosphorylation of IRE1α and XBP1 mRNA splicing yet did not affect the phosphorylation of PERK and ATF6 cleavage in the LPS-induced UPR pathway. Meanwhile, intracellular ROS production in LPS-induced macrophages increased after the knockdown of IgM. The results suggested that IgM maybe promotes Bip binding to IRE-1 to inhibit XBP1 mRNA splicing. However, the detailed mechanism of how IgM maintains ER homeostasis through binding with Bip in the ER of macrophage is unclear, and further investigation needs to be performed.

In conclusion, our results provided evidence for the existence of IgM in mice macrophages under homeostasis and RAW264.7 cell line and further illustrated that the macrophage-derived IgM V_H_DJ_H_ gene rearrangements showed a restricted recombination pattern and are individual-specific. Furthermore, we found that IgM regulated LPS-induced inflammatory response and UPR activation through interacting with Bip in macrophages. To our knowledge, this is the first-ever study to reveal the physiological function of IgM in macrophages and to renew the understanding of the biological significance of macrophage-derived IgM.

## Figures and Tables

**Table 1:** The sequences of PCR primers were used in this study.

Gene Name		Primer Sequence 5′-3′
IgM variable region	sense primer (P1)	CTTCCGGAATTCSARGTNMAGCTGSAGSAGTCWGG
	external antisense primer (P2)	CTTCAAGAAGGTGAGACCC
	internal antisense primer (P3)	GACATTTGGGAAGGACTGACTCTC
IgM constant region	sense primer (P4)	GCTAAAGGATGGGAAGCT
	antisense primer (P5)	GGATGCTGTGGGTAAAGT
F4/80	sense primer	AAGTGACTCACCTTGTGGTCC
	antisense primer	TAGATGCAAAGCCAGGGTGG
CD19	sense primer	AAGGAAGCGAATGACTGACCC
	antisense primer	GAAGTCCATCATCCTGCCA
β-actin	sense primer	CAGGGTGTGATGGTGGGAAT
	antisense primer	GGAAAAGAGCCTCAGGGCAT
IL-6	sense primer	GCCTTCTTGGGACTGATGCT
	antisense primer	GCCACTCCTTCTGTGACTCC
MCP-1	sense primer	GGCTCAGCCAGATGCAGTTA
	antisense primer	GTGCTTGAGGTGGTTGTGGA
IL-1β	sense primer	TGCCACCTTTTGACAGTGAT
	antisense primer	TGGGTGTGCCGTCTTTCATT
TNF-α	sense primer	ACGTGGAACTGGCAGAAGAG
	antisense primer	TGAGGGTCTGGGCCATAGAA
MIP-1α	sense primer	ACTGCCCTTGCTGTTCTTCT
	antisense primer	GTCTCTTTGGAGTCAGCGCA
iNOS	sense primer	GTGGTGACAAGCACATTTGG
	antisense primer	GGCTGGACTTTTCACTCTGC
XBP-1	sense primer	GAACCAGGAGTTAAGAACACG
	antisense primer	AGGCAACAGTGTCAGAGTCC

**Table 2:** The total number of V_H_DJ_H_ recombination sequences and productive sequences were analyzed for each sample.

Sample	Total Sequences	Productive Sequences	Productive Percentage (%)
No.1 WT-BMDM	3843	3704	96.38
No.2 WT-BMDM	2380	2320	97.48
No.3 WT-BMDM	2688	2596	96.58
No.1 μMT-BMDM	842	816	96.91
No.2 μMT-BMDM	929	845	90.96
No.3 μMT-BMDM	1553	1514	97.49
RAW264.7	8295	7946	95.79
No.1 WT-B	1930	1773	91.97
No.2 WT-B	2022	1827	90.36
No.3 WT-B	2085	1943	93.19

## Data Availability

The data that support the findings of this study are available from the corresponding author upon reasonable request.

## References

[1] Blandino R., Baumgarth N. (2019). Secreted IgM: New tricks for an old molecule. J. Leukoc. Biol..

[2] Sathe A., Cusick J.K. (2021). Biochemistry, Immunoglobulin M. StatPearls.

[3] Boes M. (2000). Role of natural and immune IgM antibodies in immune responses. Mol. Immunol..

[4] Haas I.G., Wabl M. (1983). Immunoglobulin heavy chain binding protein. Nature.

[5] Anelli T., Ceppi S., Bergamelli L., Cortini M., Masciarelli S., Valetti C., Sitia R. (2007). Sequential steps and checkpoints in the early exocytic compartment during secretory IgM biogenesis. EMBO J..

[6] Colucci M., Stockmann H., Butera A., Masotti A., Baldassarre A., Giorda E., Petrini S., Rudd P.M., Sitia R., Emma F. (2015). Sialylation of N-linked glycans influences the immunomodulatory effects of IgM on T cells. J. Immunol..

[7] Qiu X., Zhu X., Zhang L., Mao Y., Zhang J., Hao P., Li G., Lv P., Li Z., Sun X. (2003). Human epithelial cancers secrete immunoglobulin g with unidentified specificity to promote growth and survival of tumor cells. Cancer Res..

[8] Babbage G., Ottensmeier C.H., Blaydes J., Stevenson F.K., Sahota S.S. (2006). Immunoglobulin heavy chain locus events and expression of activation-induced cytidine deaminase in epithelial breast cancer cell lines. Cancer Res..

[9] Hu F., Zhang L., Zheng J., Zhao L., Huang J., Shao W., Liao Q., Ma T., Geng L., Yin C.C. (2012). Spontaneous production of immunoglobulin M in human epithelial cancer cells. PLoS ONE.

[10] Shao W., Hu F., Ma J., Zhang C., Liao Q., Zhu Z., Liu E., Qiu X. (2016). Epithelial cells are a source of natural IgM that contribute to innate immune responses. Int. J. Biochem. Cell Biol..

[11] Nourshargh S., Alon R. (2014). Leukocyte migration into inflamed tissues. Immunity.

[12] Wynn T.A., Chawla A., Pollard J.W. (2013). Macrophage biology in development, homeostasis and disease. Nature.

[13] Wu Y., Hirschi K.K. (2020). Tissue-Resident Macrophage Development and Function. Front. Cell Dev. Biol..

[14] Locati M., Curtale G., Mantovani A. (2020). Diversity, Mechanisms, and Significance of Macrophage Plasticity. Annu. Rev. Pathol..

[15] Netea M.G., Balkwill F., Chonchol M., Cominelli F., Donath M.Y., Giamarellos-Bourboulis E.J., Golenbock D., Gresnigt M.S., Heneka M.T., Hoffman H.M. (2017). A guiding map for inflammation. Nat. Immunol..

[16] Fuchs T., Hahn M., Ries L., Giesler S., Busch S., Wang C., Han J., Schulze T.J., Puellmann K., Beham A.W. (2018). Expression of combinatorial immunoglobulins in macrophages in the tumor microenvironment. PLoS ONE.

[17] Busch S., Talamini M., Brenner S., Abdulazim A., Hanggi D., Neumaier M., Seiz-Rosenhagen M., Fuchs T. (2019). Circulating monocytes and tumor-associated macrophages express recombined immunoglobulins in glioblastoma patients. Clin. Transl. Med..

[18] Huang J., Sun X., Gong X., He Z., Chen L., Qiu X., Yin C.C. (2014). Rearrangement and expression of the immunoglobulin mu-chain gene in human myeloid cells. Cell. Mol. Immunol..

[19] Qiu X., Sun X., He Z., Huang J., Hu F., Chen L., Lin P., You M.J., Medeiros L.J., Yin C.C. (2013). Immunoglobulin gamma heavy chain gene with somatic hypermutation is frequently expressed in acute myeloid leukemia. Leukemia.

[20] Shao W., Zhang C., Liu E., Zhang L., Ma J., Zhu Z., Gong X., Qin Z., Qiu X. (2016). Identification of Liver Epithelial Cell-derived Ig Expression in mu chain-deficient mice. Sci. Rep..

[21] Brezinschek H.P., Foster S.J., Brezinschek R.I., Dorner T., Domiati-Saad R., Lipsky P.E. (1997). Analysis of the human VH gene repertoire. Differential effects of selection and somatic hypermutation on human peripheral CD5(+)/IgM+ and CD5(-)/IgM+ B cells. J. Clin. Investig..

[22] Ward C., Rettig T.A., Hlavacek S., Bye B.A., Pecaut M.J., Chapes S.K. (2018). Effects of spaceflight on the immunoglobulin repertoire of unimmunized C57BL/6 mice. Life Sci. Space Res..

[23] Owen K.A., Pixley F.J., Thomas K.S., Vicente-Manzanares M., Ray B.J., Horwitz A.F., Parsons J.T., Beggs H.E., Stanley E.R., Bouton A.H. (2007). Regulation of lamellipodial persistence, adhesion turnover, and motility in macrophages by focal adhesion kinase. J. Cell Biol..

[24] Preissler S., Ron D. (2019). Early Events in the Endoplasmic Reticulum Unfolded Protein Response. Cold Spring Harb. Perspect. Biol..

[25] Todd D.J., Lee A.H., Glimcher L.H. (2008). The endoplasmic reticulum stress response in immunity and autoimmunity. Nat. Rev. Immunol..

[26] Kopp M.C., Larburu N., Durairaj V., Adams C.J., Ali M.M.U. (2019). UPR proteins IRE1 and PERK switch BiP from chaperone to ER stress sensor. Nat. Struct. Mol. Biol..

[27] Bornemann K.D., Brewer J.W., Beck-Engeser G.B., Corley R.B., Haas I.G., Jack H.M. (1995). Roles of heavy and light chains in IgM polymerization. Proc. Natl. Acad. Sci. USA.

[28] Malhotra J.D., Kaufman R.J. (2007). Endoplasmic reticulum stress and oxidative stress: A vicious cycle or a double-edged sword?. Antioxid. Redox Signal..

[29] Wang C., Xia M., Sun X., He Z., Hu F., Chen L., Bueso-Ramos C.E., Qiu X., Yin C.C. (2015). IGK with conserved IGKappaV/IGKappaJ repertoire is expressed in acute myeloid leukemia and promotes leukemic cell migration. Oncotarget.

[30] Mandric I., Rotman J., Yang H.T., Strauli N., Montoya D.J., Van Der Wey W., Ronas J.R., Statz B., Yao D., Petrova V. (2020). Profiling immunoglobulin repertoires across multiple human tissues using RNA sequencing. Nat. Commun..

[31] Jiang D., Ge J., Liao Q., Ma J., Liu Y., Huang J., Wang C., Xu W., Zheng J., Shao W. (2015). IgG and IgA with potential microbial-binding activity are expressed by normal human skin epidermal cells. Int. J. Mol. Sci..

[32] Tang J., Zhang J., Liu Y., Liao Q., Huang J., Geng Z., Xu W., Sheng Z., Lee G., Zhang Y. (2018). Lung squamous cell carcinoma cells express non-canonically glycosylated IgG that activates integrin-FAK signaling. Cancer Lett..

[33] Zhang C., Xiao L., Huang Y., Zhang L., Jiang D., Shao W., Zheng J., Hu F., Chu M., Huang J. (2021). NBIGV-DB: A dedicated database of non-B cell derived immunoglobulin variable region. Gene.

[34] Maa M.C., Chang M.Y., Chen Y.J., Lin C.H., Yu C.J., Yang Y.L., Li J., Chen P.R., Tang C.H., Lei H.Y. (2008). Requirement of inducible nitric-oxide synthase in lipopolysaccharide-mediated Src induction and macrophage migration. J. Biol. Chem..

[35] Mitra S.K., Hanson D.A., Schlaepfer D.D. (2005). Focal adhesion kinase: In command and control of cell motility. Nat. Rev. Mol. Cell Biol..

[36] Yang M., Zhang F., Qin K., Wu M., Li H., Zhu H., Ning Q., Lei P., Shen G. (2016). Glucose-Regulated Protein 78-Induced Myeloid Antigen-Presenting Cells Maintained Tolerogenic Signature upon LPS Stimulation. Front. Immunol..

[37] Feige M.J., Groscurth S., Marcinowski M., Shimizu Y., Kessler H., Hendershot L.M., Buchner J. (2009). An unfolded CH1 domain controls the assembly and secretion of IgG antibodies. Mol. Cell.

[38] Hetz C., Glimcher L.H. (2009). Fine-tuning of the unfolded protein response: Assembling the IRE1alpha interactome. Mol. Cell.

[39] Hetz C., Papa F.R. (2018). The Unfolded Protein Response and Cell Fate Control. Mol. Cell.

[40] Adams C.J., Kopp M.C., Larburu N., Nowak P.R., Ali M.M.U. (2019). Structure and Molecular Mechanism of ER Stress Signaling by the Unfolded Protein Response Signal Activator IRE1. Front. Mol. Biosci..

[41] Martinon F., Chen X., Lee A.H., Glimcher L.H. (2010). TLR activation of the transcription factor XBP1 regulates innate immune responses in macrophages. Nat. Immunol..

